# Audiovisual Association Learning in the Absence of Primary Visual Cortex

**DOI:** 10.3389/fnhum.2015.00686

**Published:** 2016-01-05

**Authors:** Mehrdad Seirafi, Peter De Weerd, Alan J. Pegna, Beatrice de Gelder

**Affiliations:** ^1^Department of Cognitive Neuroscience, Faculty of Psychology and Neuroscience, Maastricht UniversityMaastricht, Netherlands; ^2^Donders Institute for Brain, Cognition and Behaviour, Radboud UniversityNijmegen, Netherlands; ^3^Department of Neurology, Geneva University HospitalGeneva, Switzerland

**Keywords:** audiovisual learning, superior colliculus, blindsight

## Abstract

Learning audiovisual associations is mediated by the primary cortical areas; however, recent animal studies suggest that such learning can take place even in the absence of the primary visual cortex. Other studies have demonstrated the involvement of extra-geniculate pathways and especially the superior colliculus (SC) in audiovisual association learning. Here, we investigated such learning in a rare human patient with complete loss of the bilateral striate cortex. We carried out an implicit audiovisual association learning task with two different colors of red and purple (the latter color known to minimally activate the extra-genicular pathway). Interestingly, the patient learned the association between an auditory cue and a visual stimulus only when the unseen visual stimulus was red, but not when it was purple. The current study presents the first evidence showing the possibility of audiovisual association learning in humans with lesioned striate cortex. Furthermore, in line with animal studies, it supports an important role for the SC in audiovisual associative learning.

## Introduction

There is strong evidence from neurophysiological animal experiments for the confluence of visual and auditory cues in superior colliculus (SC) (Jay and Sparks, 1984; Meredith and Stein, 1986; Alvarado et al., 2007; Yu et al., 2010). This suggests a major role in audiovisual association-based learning. To test this idea, the role of the geniculo-cortical pathway can be limited (thus increasing the role of the extra-genicular pathway) by ablating the striate cortex in animals. This has been done in the cat (Jiang et al., 2015), but due to the strong retino-genicular projections to extrastriate cortex (Rosenquist, 1985), it is difficult in such experiments to attribute audiovisual learning to extra-genicular pathways and, specifically, the SC. In monkeys, lateral geniculate nucleus (LGN) targets the primary visual cortex (V1) almost exclusively (Sincich and Horton, 2005), such that a V1 lesion will render the animal blind, and make any residual visual capacities (Humphrey, 1974) and capacities for audiovisual learning, dependent on extra-genicular pathways and nuclei, including the SC.

A rare patient (TN) with a complete bilateral lesion of striate cortex gave us the opportunity to study potential contributions of the SC to human audiovisual learning in the absence of the retino-geniculo-cortical pathway. Establishing audiovisual learning in hemianopic patients and understanding the underlying mechanisms is important in view of the potential for audiovisual learning in such patients which can improve self-reliance.

The indication that there is sufficient visual information being processed that could be associated with normally perceived auditory information led to the idea to study audiovisual learning in an individual with a striate cortex lesion. Although damage to striate cortex severally disrupts normal visual processes causing blindness (i.e., the absence of conscious visual perception), some patients show remarkable residual visual abilities as evidenced by above-chance detection or discrimination of movement (Barbur et al., 1980), wavelength (Stoerig and Cowey, 1989), flicker (Blythe et al., 1987), orientation (Weiskrantz et al., 1974), motion direction (Pizzamiglio et al., 1984), and object shape (Weiskrantz et al., 1974). The extra-genicular retino-cortical pathway is a possible substrate underlying residual, visually driven behavior (Weiskrantz et al., 1974). The SC is the earliest post-retinal structure of this pathway, and remarkably, does not receive direct input from the s-cones (Schiller and Malpeli, 1977; Leh et al., 2006; Marzi et al., 2009; Tamietto et al., 2010). Because of the important role of the SC in audiovisual integration (Meredith and Stein, 1986), we hypothesized a contribution of SC in audiovisual association learning, which we believe to be exclusive for colored stimuli in medium-to-long wavelengths. These ideas led us to test two predictions in a new implicit audiovisual association task, in which some auditory target stimuli were predictable on the basis of associated unseen visual information. We predicted first, that given the audio-visual integrative function of the SC, the cortically blind patient TN would acquire shortened reaction times (RT) to the visually predicted auditory targets, and, second, given the SC’s lack of sensitivity for short wavelengths (Marzi et al., 2009), that this RT advantage would be absent for visual stimuli that were colored purple.

## Materials and Methods

### Participant

**FIGURE 1:**
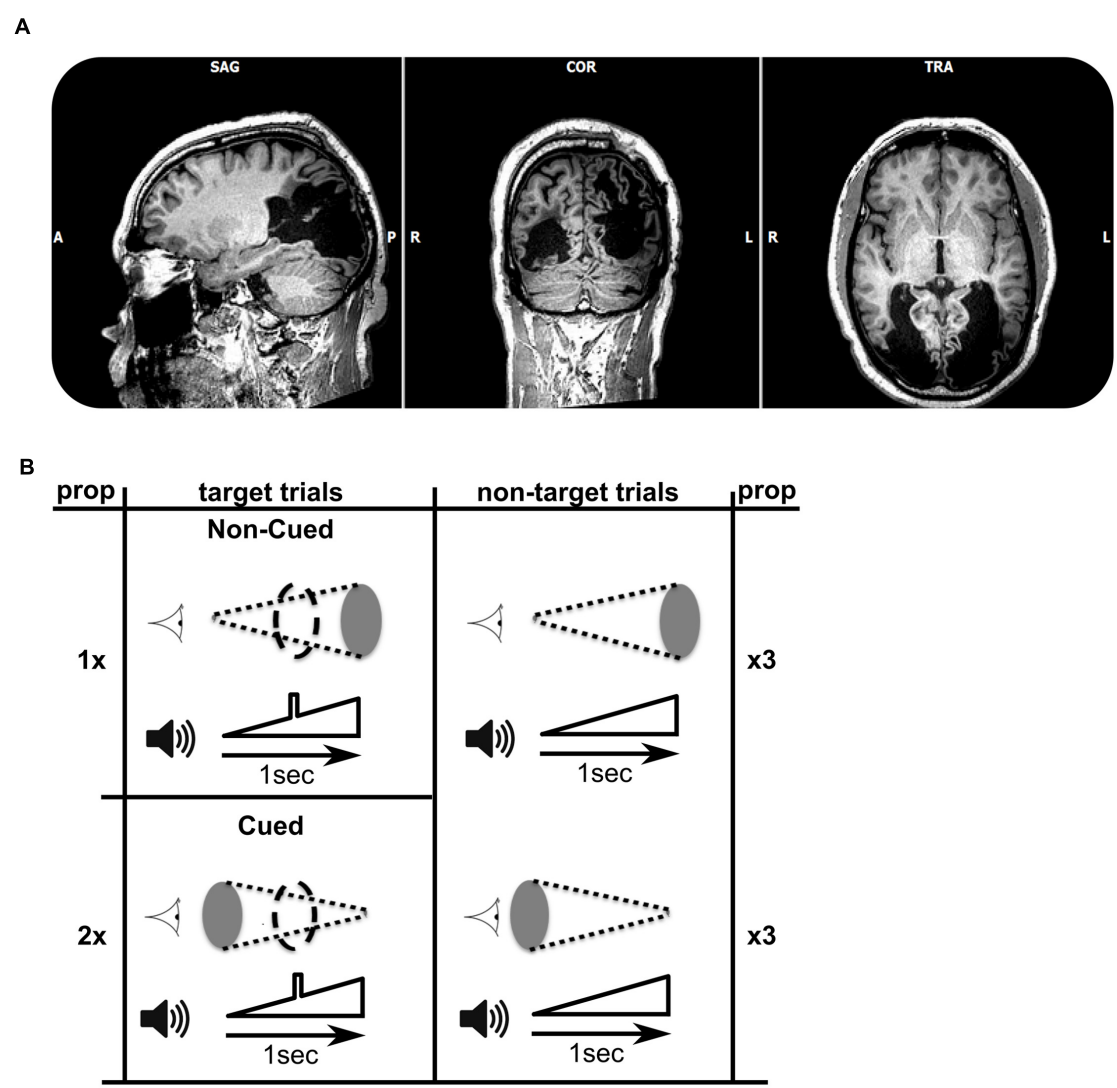
**(A)** T1-weighted MRI (axial, coronal, and saggital views) showing the extent of bilateral striate cortex lesions. In terms of Brodmann’s areas, the left hemisphere lesion encompasses BA 17, 18, 19, 37, and 39. The right hemisphere lesion encompasses BA 17, 18, and 19. Lesion sites look black as they are filled with Cerebrospinal fluid (CSF). **(B)** Schematic view of the target and non-target combinations of audiovisual stimuli. The visual stimulus is represented next to the cartoon of an eye, and consisted of a disk either increasing in size over 1 s (top row), or decreasing in size (bottom). The auditory stimulus, shown next to the speaker cartoon, always consisted of a sound of increasing volume over a period of 1 s. A subgroup of trials (left column, three of each nine trials) included an auditory target (a square-wave modulation in volume) in the middle of the 1 s trial. Stimuli were presented in cycles of nine trials, each of which contained six non-target trials and three target trials (the numbers 1, 2, and twice 3 refer to the frequencies of occurrence of trials within a single trial cycle). The data sets for each of the two experiments consisted of 30 cycles. The patient was asked to respond as fast as possible to each target. Within the target trials, the more probable audiovisual combination was called ‘cued,’ with the increasing (looming) visual stimulus cueing the presence of an auditory target. The less frequent combination was named ‘non-cued.’

The participant was a 56 year-old right-handed patient (TN) who became cortically blind bilaterally following two strokes within 6 weeks (**Figure 1A**). Previous studies documented his inability to perceive color, movement, and object shape presented in the visual modality alone, yet, he demonstrated above-chance level performance in a facial expression discrimination task (Pegna et al., 2005) and intact visual navigation skill (de Gelder et al., 2008). The participant gave informed consent in accordance with the Helsinki protocols, and all procedures were performed according to requirements of the Ethical Committee of the Faculty of Psychology and Neuroscience.

### Experimental Design and Procedure

TN was presented with an auditory looming stimulus of 1 s, during which the stimulus increased in volume (symbolized in **Figure 1B**). In a subset of trials a brief square-wave modulation in volume was given superimposed on the overall increasing intensity of the sounds. This modulation represented the target to which the patient had to respond as quickly as possible in the target trials. The target was clearly audible during the trial, and occurred in the middle of the target trials (see **Figure 1B**, left). In other trials the auditory target was absent (non-target trials). Hence, auditory target and non-target trials were identical except for the brief square-wave modulation in the middle of target-trials. One third of the trials consisted of target trials, and two thirds were non-target trials. A single experiment comprised 270 1-s-stimulus presentations, followed by a 500 ms response window, and an inter-trial interval lasting between 400 and 700 ms.

In Experiment 1, we created an audio-visual association which was predictive of the presence of the auditory target. Two red visual disk stimuli were used (symbolized by gray ellipses in **Figure 1B**), increasing in size (looming) or decreasing (receding). To ensure visual stimulation, we used a small viewing distance (∼25 cm) and gaze was monitored by two of the experimenters. The patient was not given information about the presence of any systematic associations between auditory and visual stimuli, but was informed that visual stimuli were being presented. The audiovisual pairings were designed as follows: In *non-target trials* (*n* = 180), half of the trials were associated with simultaneously receding visual stimuli, and the other half were associated with looming visual stimuli. However, among *auditory target trials* (*n* = 90), two-thirds of the trials (*n* = 60) paired the auditory stimulus with a receding visual signal, and only one-third (*n* = 30) paired the auditory stimulus with a looming visual signal. Therefore, in the overall distribution of audio-visual pairings, the presence of a receding visual stimulus is predictive of an auditory target (*cued target*). Thus, the auditory target is cued by the receding visual stimulus, because the auditory target is paired more frequently with the receding visual stimulus than with the looming visual stimulus. To maintain the parallelism of visual and auditory stimuli, we included a visual target analog in the visually looming or receding stimuli, paired with auditory targets. The visual target consisted of a maximally sized disk that briefly interrupted the smoothly varying size of the disk (indicated by coarse dashed outline in **Figure 1B**). Experiment 2 consisted of a repetition of Experiment 1 with purple visual stimuli instead of red.

Note that in the two experiments, the cueing of the auditory target stimulus occurred by a higher-probability pairing between the target and a receding visual stimulus. Hence, the looming auditory sound present in all trials was incongruent with the receding visual stimulus. We were most interested in cueing the auditory stimulus with the incongruent visual stimulus, as we wished to study the effect of learning an association (or a predictive relation) without a possible confounding advantage due to congruency. If we had chosen a congruent pairing, levels of performance may have reflected contributions from both a congruency effect and a learning effect. The chosen cueing strategy therefore enabled us to study a more pure effect of learning.

### Stimulus Preparation

At the patient’s position, the background noise was 35 dB, and the auditory looming stimulus was added on top of the noise, increasing from 35 dB to a maximum of 80 dB. The noise (measured in the patient’s position) was due to ambient noise in the room due to computers and other equipment. The patient was seated in a dark room at a distance of approximately 25 cm from the monitor (0.2 cd/m2) and a chin rest supported his head. The audiovisual stimuli were prepared as follows: first, an auditory wave of 500 Hz looming sound of 1 s was generated with and without target sounds, using GoldWave v.5.68 (GoldWave, Inc.). The target sound was generated by inserting the maximum amplitude in the middle of the looming wave time course (in the middle of the stimulus at *t* = 500 ms, duration = 10 ms, loudness = 80 dB).

Next, looming and receding videos were produced in Adobe Flash CS6 (www.adobe.com) and synchronized with the auditory waves resulting in four different video files (60 Frame/s) for each of the red and purple colors used [red (colorimetric values: *x* = 0.60, *y* = 0.35; luminance = 34 cd/m2); purple (*x* = 0.40, *y* = 0.24; luminance = 35 cd/m2)]. At the viewing distance used, the visual looming stimulus varied from 4.8° to 67° diameter. The visual receding stimulus was exactly the same as the looming stimulus, only with opposite order of frames in time. Target analogs in looming and receding visual stimuli corresponded to a replacement of the middle frame (31st frame) by a frame containing the largest disk (67°).

To ensure that the purple color was in the wavelength in which the SC is insensitive, we used an established, indirect method (Tamietto et al., 2010), in seven normal participants in a control experiment. Participants were seated at 57 cm from the screen on which the stimuli were presented. The basic idea of the control experiment is that SC is assumed to provide an RT advantage to bilateral visual targets compared to unilateral targets when stimuli are red, but not when they are purple (given the insensitivity for purple in SC). We used the red and purple colors that were used in our experiment and applied them to the paradigm used by Tamietto et al. (2010), as follows: Red or purple target stimuli (small squares of 2.5° × 2.5°) were projected for 200 ms against a background consisting of other achromatic squares (2.5°) that were changing luminance every 50 ms (range: 1.1–60 cd/m2). The background was in view for a variable period prior to the onset of the target(s). Target stimuli were presented on the horizontal meridian to one or both sides of the fixation point (ISI = 3600–4300 ms), at a distance of 5° from a central fixation point. There were five randomly intermingled stimulus conditions: single and double red target stimuli, single and double purple target stimuli, and trials without any target stimulus (catch trials). The task of the control participants was to press a button as fast as possible after seeing any stimulus appear on the screen.

## Results

**FIGURE 2:**
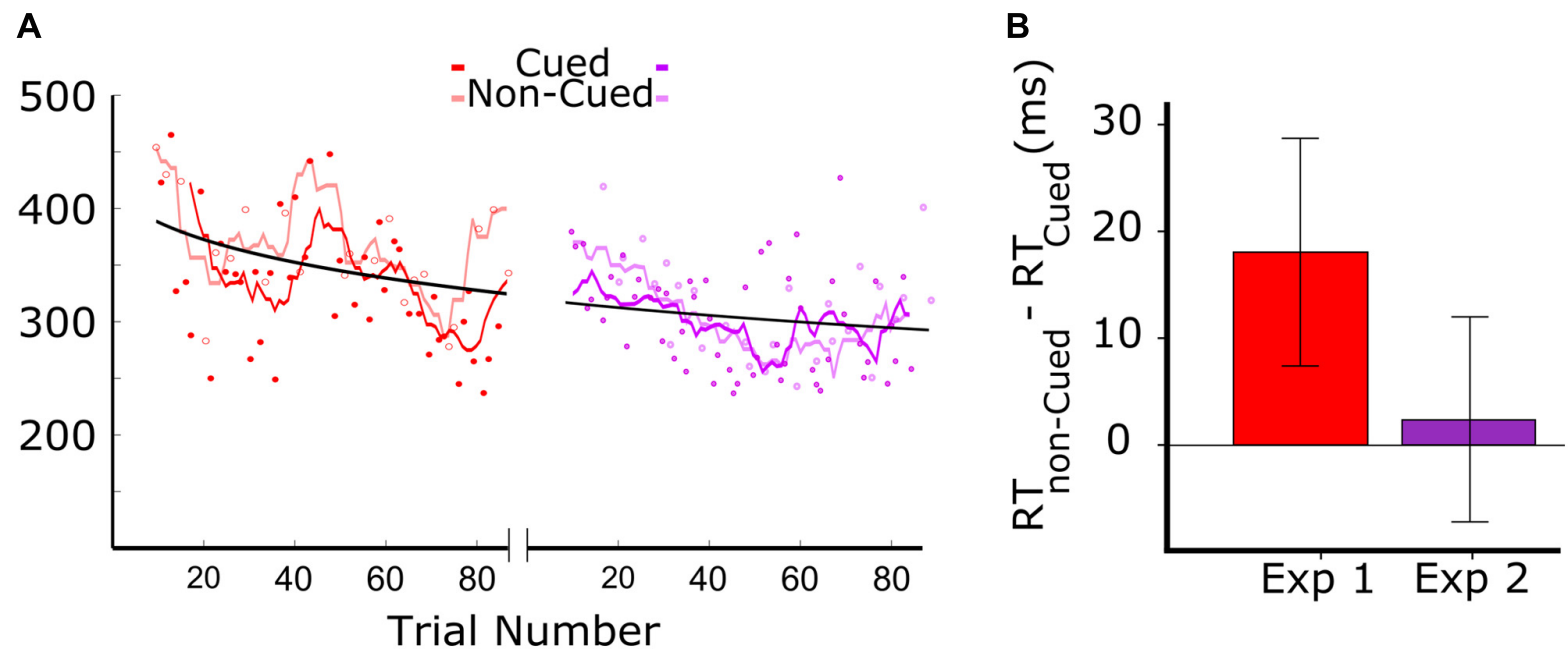
**Results of Experiments 1 and 2.** Red and purple colors are associated to Experiments 1 and 2, where the color of the visual stimuli were red and purple, respectively **(A)** Reaction times (RT) as a function of target trial number in Experiments 1 and 2. In Experiment 1, dark red and light red symbols represent cued and non-cued trials, respectively. The dark red line represents the smoothed RTs of cued trials, and the light red line shows the smoothed RTs of non-cued trials (length of the moving average window = 3 cycles). For the representation of data from Experiment 2, analogous conventions were followed. **(B)** Difference between the RT in cued and non-cued conditions for the two experiments. Both bars show the RT difference between cued and non-cued trials averaged over all cycles. The error-bars indicate the 95% confidence intervals.

**Figure 2** shows the results from the two experiments. **Figure 2A** (left panel) shows the data for audiovisual learning in Experiment 1, with the darker red trend line corresponding to RTs to auditory targets that were associated with the visual cue, and the lighter red trend line corresponding to the RTs to auditory targets that were not associated with the visual cue. The trend lines were generated by a moving average including a window of six trials (moved with a step size of one trial). The first 18 trials were excluded from analysis as the patient didn’t respond within the 500 ms response window. Of the 90 auditory target trials administered in Experiment 1, we therefore kept 44 trials with visually cued auditory targets, and 24 trials with non-cued targets. RTs above 500 were excluded, resulting in the first 20 trials of Experiment 1 not being included. The data clearly show a systematic tendency of the RTs on cued trials (dark red) to be lower than those on non-cued trials (light red).

The data in **Figure 2A**, combined for the two experiments (red: Experiment 1; purple: Experiment 2), show an overall reduction in RT that fit well in a power law curve. This suggests an aspecific learning effect, with the patient becoming more skilled in responding to the auditory target.

Prior to testing the differences between cued and non-cued conditions, we verified whether the distribution of RT differences was Gaussian. A Kolmogorov–Smirnov test showed for both datasets that normality was not rejected (red: *D* = 0.14, *p* = 0.20; purple: *D* = 0.16, *p* = 0.16). This allowed us to apply a paired *t*-test. Cued and non-cued trials were paired based on trial-cycle, with each consecutive cycle of nine trials containing all trial types, including the three cued and non-cued trials. As shown in **Figure 2B**, we found a significant RT advantage for the cued over the non-cued condition in Experiment 1 [*t*(23) = 3.98; *p* = 0.001], but no significant difference between the two conditions in Experiment 2 [*t*(27) = 1.65; *p* = 0.11].

### Control Experiment

To verify that the colors used in our experiment would show the properties expected for contribution (red) or non-contribution (purple) from the SC, we performed a control experiment, based on the idea that RTs in a speeded RT test to two targets shown bilaterally are faster than to a single target shown unilaterally. It has been shown that this RT advantage disappears when stimuli are made purple (Marzi et al., 2009), and hence we replicated this experiment with the specific colors used in our experiment (details in Materials and Methods). A paired sample *t*-test showed significant reduction in RT for the bilateral condition compared to the unilateral one for red stimuli but not for the purple ones [seven participants, red: *t*(6) = 3.1, *p* < 0.05; purple: *t*(6) = 0.99, *p* = 0.36], suggesting that the purple color used in our experiment would indeed lead to a reduction in the contribution of SC to audiovisual association learning.

## Discussion

The result from Experiment 1 shows that the patient with complete striate damage can implicitly learn the association between the auditory and the visual stimuli when the stimuli are colored red. We demonstrated in Experiment 2 that the patient can no longer make use of the learned association when the stimuli are rendered purple.

The present study is the first demonstrating the fast development of implicit audiovisual associations (over the course of just a few 10s of trials) between consciously perceived auditory stimuli and visual stimuli that cannot be perceived because of complete bilateral striate lesion. The finding that the implicit audiovisual association did not take place for the purple visual stimuli strongly supports the notion that SC plays a critical role in this form of learning. A control experiment in seven normal participants (details in Materials and Methods) showed a RT advantage when responding to two red, compared to one red target in the visual field; an advantage that disappeared with purple stimuli. As the SC is thought to contribute to this RT advantage, these findings support the idea that the purple color used in our visual stimuli indeed could not be processed by the SC (Tamietto et al., 2010).

Our data are in line with demonstrations of automatic neural audiovisual integration in the anesthetized cat (Yu et al., 2010) and with the anatomical loop between SC and basal ganglia (Redgrave et al., 2010), which may facilitate rapid implicit learning. Furthermore, our result is also consistent with the data from the only other bilateral cortically blind patient studied, who exhibited effective conditioning of startle responses to visual stimuli (Hamm et al., 2003). That finding established another form of implicit learning involving unseen stimuli, likely to depend on extra-genicular visual projections to amygdala and cerebellum (Morris et al., 1998; Timmann et al., 2010). The cessation in our study of audiovisual association learning for purple stimuli is consistent with previous research showing the role of SC in unilateral cortically blind patients in various residual visual abilities (Marzi et al., 2009). Our study shows that the extra-genicular pathways not only underlies the residual capacities for visual processing outside awareness, but also contributes to the implicit associative learning between visual stimuli and other relevant information. This opens new perspectives for rehabilitation in cortically blind patients (Boyd and Winstein, 2006).

## Conflict of Interest Statement

The authors declare that the research was conducted in the absence of any commercial or financial relationships that could be construed as a potential conflict of interest.
